# 

**Published:** 1886-04

**Authors:** 


### CONTENTS.

LIFE AND CHARACTER of Dr. Ashbel Smith .......... 440

CORRESPONDENCE—Our New York Letter—The Mechanical Treatment of Hernia........................ 455

SOCIETY NOTES—Letter from Dr. Burt, Sec. T. S. M. A.—Bell County Medical Society, Minutes, etc.—Programme 18th Annual Convention T. S M. A.—No Reduction in Fare—Cass County Medical Society—Burleson County Medical Society—Long Medical Society ................................ 462–467

EDITORIAL—How Medical Journalism has Aided the Profession in Texas—A Much Needed Work...... 468–469

EDITORIAL NOTES—Texas Surgery—A Merited Compliment—The Late Ashbel Smith—Minor Paragraphs—The Presidency of the Congress—Bacillus Decalvans—Texas Quarantine—Such is Fame—The Wrong Track—Next—Reminiscences—Wanted—A Grub to Catch Gudgeons...................... 476–480

NECROLOGICAL—Dr. G. K. McGregor................. 481

ADVERTISERS' NOTICES............................. 482–484

